# Extraction of *Ficus carica* Polysaccharide by Ultrasound-Assisted Deep Eutectic Solvent-Based Three-Phase Partitioning System: Process Optimization, Partial Structure Characterization, and Antioxidant Properties

**DOI:** 10.3390/molecules30173469

**Published:** 2025-08-23

**Authors:** Qisen Sun, Zhubin Song, Fanghao Li, Xinyu Zhu, Xinyu Zhang, Hao Chen

**Affiliations:** 1 SDU-ANU Joint Science College, Shandong University, Weihai 264209, China; sunqs@mail.sdu.edu.cn (Q.S.); 202200700196@mail.sdu.edu.cn (Z.S.); 202200700176@mail.sdu.edu.cn (F.L.); 202200700229@mail.sdu.edu.cn (X.Z.); 2 Marine College, Shandong University, Weihai 264209, China; 17835855549@163.com

**Keywords:** deep eutectic solvents, dodecanoic acid, three-phase partitioning, bioactive polysaccharides, recyclable extractant, response surface methodology, antioxidant activity

## Abstract

An innovative ultrasound-assisted deep eutectic solvent-based three-phase partitioning (UA-DES-TPP) system was developed for the sustainable extraction of *Ficus carica* polysaccharide (FCP). Using a hydrophobic DES composed of dodecanoic acid and octanoic acid (1:1 molar ratio), a phase behavior-driven separation mechanism was established. The system was systematically optimized through single-factor experiments and response surface methodology (RSM), achieving a maximum FCP yield of 9.22 ± 0.20% under optimal conditions (liquid–solid ratio 1:24.2 g/mL, top/bottom phase volume ratio 1:1.05 *v*/*v*, ammonium sulfate concentration 25.8%). Structural characterization revealed that FCP was a heteropolysaccharide primarily composed of glucose and mannose with α/β-glycosidic linkages and a loose fibrous network. Remarkably, the DESs demonstrated excellent recyclability over five cycles. Furthermore, FCP exhibited significant concentration-dependent antioxidant activities: 82.3 ± 3.8% DPPH radical scavenging at 8 mg/mL, 76.8 ± 0.8% ABTS^+^ scavenging, and ferric ion reducing power of 45.53 ± 1.07 μmol TE/g. This study provides a new path for the efficient and sustainable extraction of bioactive macromolecules.

## 1. Introduction

*Ficus carica* (*F. carica*), belonging to the Moraceae plant, is native to Western Asia and the eastern Mediterranean region [1,2]. *F. carica* is suitable for cultivation in the Mediterranean climate zone or in the subtropical and temperate regions around the world [3]. *F. carica* is widely distributed in Xinjiang, Shandong, Sichuan, and other places in China [4,5]. The *F. carica* fruit contains various bioactive substances such as polysaccharides, organic acids, flavonoid compounds, phenolic compounds, vitamins, and trace elements, showing important nutritional and medicinal values [6]. Studies have shown that these bioactive components play a positive role in anti-cancer, anti-inflammatory, antioxidant, antibacterial, metabolic regulation, and prevention of chronic disease, thus attracting wide attention [7,8]. In view of its unique flavor, nutritional value, and potential health benefits, the deep processing and industrial development of *F. carica* are increasingly valued in the global food and health fields [9].

In the components of *F. carica*, the reserve of polysaccharides is relatively abundant [10]. FCP has a variety of excellent biological activities, such as outstanding anti-inflammatory, antibacterial, antioxidant, anti-tumor, and immunomodulatory properties, etc., which make it widely used in many fields such as pharmaceuticals, cosmetics, health products, and functional foods [11,12]. Extraction technology plays a crucial role in the development and application of natural polysaccharides. The traditional hot water extraction method (HWE) is characterized by safety and low cost, and it is a commonly used method for polysaccharide extraction [13,14]. However, this method has low extraction efficiency and high impurity content, and the polysaccharide structure is easily damaged in a high-temperature environment [15]. With the development of technology, it has triggered an innovation in polysaccharide extraction methods. More and more auxiliary extraction technologies, such as ultrasonic, microwave, freeze–thaw, enzyme, etc., are applied to the extraction of natural polysaccharides, effectively solving the problem of low efficiency of HWE [16,17,18]. In addition to these methods, dilute acids/bases or organic solvents are also used for the extraction of polysaccharides [19]. However, these methods may damage the structure and biological activity of polysaccharides and pose safety hazards in production, and improper treatment of waste liquid can also cause environmental pollution [20]. Among these methods, the enzyme-assisted extraction method is the greenest and mildest one [21]. However, the enzyme extraction method has problems such as enzyme residue, instability, and high cost, which hinder its further application [22,23]. Therefore, it is urgent to find an efficient and environmentally friendly method for extracting natural polysaccharides.

Three-phase partitioning (TPP) is a highly promising and efficient biological separation technique, which is favored for its simplicity of operation, high cost-effectiveness, and environmental friendliness [24]. This technique induces the formation of a distinct three-phase system by adding specific inorganic salts and organic solvents to the crude extract: the upper organic phase mainly enriches low-molecular-weight pigments or lipids; the middle layer is the protein precipitation phase; while the lower aqueous phase retains highly polar components such as polysaccharides [25]. This unique stratification mechanism enables it to efficiently separate key bioactive molecules, such as proteins, enzymes, polysaccharides, and oils, in one step directly from complex mixtures. Although TPP demonstrates excellent scalability and broad application potential under mild conditions, the commonly used solvent tert-butanol, in current use, poses significant challenges, including volatility, flammability, and toxicity [26]. Therefore, developing green and safe alternative solvents is an important research direction to promote the wider application of TPP technology.

Deep eutectic solvents (DESs), as an emerging green solvent system, have received extensive attention in recent years due to their excellent environmental protection characteristics and versatility [27]. DESs are usually formed by the combination of a hydrogen bond acceptor (HBA) and a hydrogen bond donor (HBD) through intermolecular hydrogen bonding [28]. Their preparation methods, such as heating, stirring, and grinding, are simple and low-cost. Compared with traditional organic solvents, DESs (dodecanic acid-octanoic acid system) exhibit significant advantages, such as low or non-toxicity, low volatility, biodegradability, good recyclability, and excellent solubility [29]. They can effectively dissolve various substances with different structures and properties, including polysaccharides, proteins, polyphenols, flavonoids, and alkaloids, thus preserving the biological activity of the extracts [30,31]. These characteristics make DESs an ideal substitute for tert-butanol, providing a green solution to the problems of volatile, flammable, and toxic solvents faced in the TPP technology [32]. In addition, the low melting point of DESs often makes it liquid at room temperature, promoting their full contact with materials and improving the extraction efficiency [33]. Therefore, the strategic integration of DESs with TPP technology has opened up an innovative path for the efficient and environmentally friendly separation and purification of natural bioactive components, especially polysaccharides [34,35,36].

Therefore, this study aims to develop an ultrasound-assisted DES-based TPP system for the extraction and purification of polysaccharides from *Ficus carica*. Single-factor experiments were conducted in the study, including the mass fraction of (NH_4_)_2_SO_4_, solid–liquid ratio, volume ratio of the upper layer to the lower layer, extraction temperature, and extraction time. A TPP extraction method most suitable for FCP was developed in combination with the response surface analysis experiment. Structural characteristic analyses such as scanning electron microscopy, Fourier transform infrared spectroscopy, and monosaccharide composition analysis were performed on the obtained *Ficus carica* polysaccharides. Finally, the thermal stability and antioxidant capacity of FCP were further investigated. This study provides a new idea for the efficient extraction and separation of plant polysaccharides using TPP.

## 2. Results and Discussion

### 2.1. Single Factor Experimental Analysis

**Figure 1:**
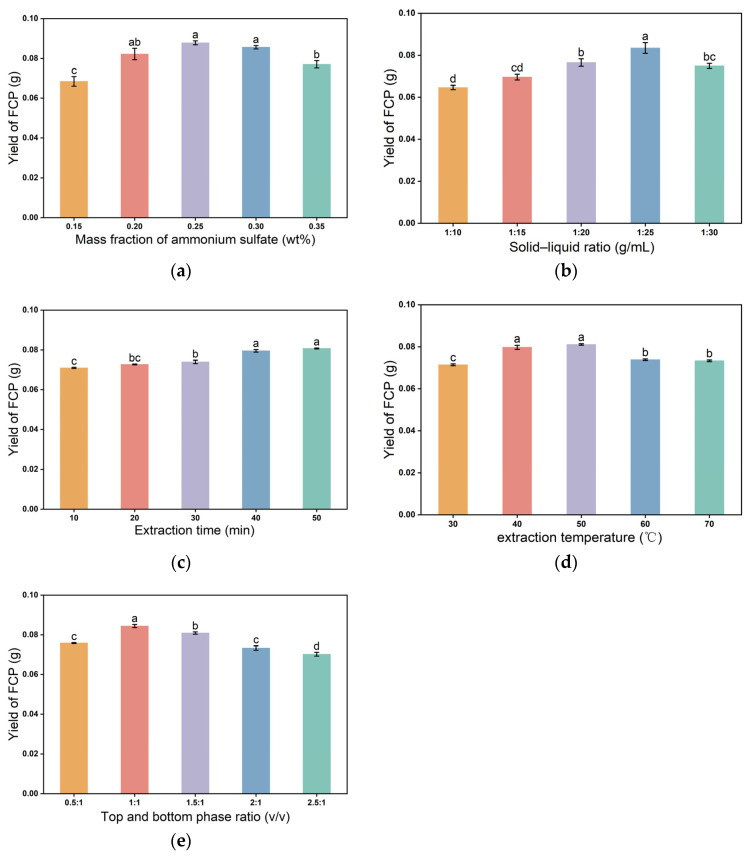
Effects of single-factor parameters on the yield of *Ficus carica* polysaccharides (FCP): (**a**) ammonium sulfate concentration (15–35 wt%, fixed ultrasonic time 30 min, temperature 50 °C, solid–liquid ratio 1:25 g/mL); (**b**) solid–liquid ratio (1:10–1:30 g/mL, fixed ultrasonic time 30 min, temperature 50 °C, ammonium sulfate concentration 25 wt%); (**c**) ultrasonic time (10–50 min, fixed temperature 50 °C, solid–liquid ratio 1:25 g/mL, ammonium sulfate concentration 25 wt%); (**d**) extraction temperature (30–70 °C, fixed ultrasonic time 30 min, solid–liquid ratio 1:25 g/mL, ammonium sulfate concentration 25 wt%); (**e**) top/bottom phase volume ratio (0.5:1–2.5:1 *v*/*v*, fixed ultrasonic time 30 min, temperature 50 °C, solid–liquid ratio 1:25 g/mL, ammonium sulfate concentration 25 wt%). The *y*-axis represents the FCP yield (g). All experiments were conducted at an ultrasonic power of 300 W.

Based on the results of single-factor experiments, the effects of ammonium sulfate concentration, liquid–solid ratio, extraction time, temperature, and top/bottom phase volume ratio on the yield of FCP were systematically studied. Each group of experiments was repeated three times, and the results are shown in Figure 1.

When the ammonium sulfate concentration was in the range of 15–35%, the yield of FCP showed a significant trend of increasing first and then decreasing, and the yield was the highest at 25% concentration (0.0878 ± 0.0014 g). This phenomenon could be attributed to the dynamic balance between the salting-out effect and ionic strength: the low concentration of ammonium sulfate (<25%) promoted protein precipitation through charge neutralization, thereby releasing more bound polysaccharides into the aqueous phase; the high concentration range (>25%) weakened the interfacial tension of DES–water phase due to excessive enhancement of ionic strength, resulting in a decrease in phase separation efficiency [37]. The stable high value (0.0867–0.0891 g) of the three sets of repeated data at 25% was in stark contrast to the yield drop (0.0845–0.0776 g) after the concentration was 30%, supporting the critical transition dominated by the charge shielding effect.

When the solid–liquid ratio was in the range of 1:10 to 1:30, the yield showed an inverted U-shaped change, and the peak was at 1:25 (0.0756 ± 0.0021 g). This trend was due to two types of competition mechanisms: when the liquid–solid ratio was low (≤1:20), the solvent volume was insufficient to limit the dissolution efficiency of polysaccharides, and the residual amount in the cell wall increased; the high liquid–solid ratio (=1:30) reduced the driving force of the concentration gradient due to the dilution of the system and dispersed the ultrasonic energy density and weakened the cavitation effect [38].

When the ultrasonic time was extended to 50 min, the yield continued to increase to 0.0807 ± 0.0003 g, reflecting the positive effect of dissolution [39]. In theory, the cavitation effect could accelerate cell wall fragmentation and polysaccharide release, but after crossing the critical time (>50 min), polysaccharide chains might be broken by mechanical shear force, resulting in a decrease in molecular weight. The experimental data did not show a downward trend within 50 min, indicating that the current system had not yet reached the degradation threshold, but the subsequent response surface needed to be combined with structural characterization to verify the stability of long-term extraction. For all single-factor experiments, unless the ultrasonic time was the variable under investigation, a fixed ultrasonic time of 30 min and ultrasonic power of 300 W were applied. This ensured consistency across experiments and allowed for accurate assessment of each individual factor.

The highest yield (0.0811 ± 0.0005 g) was obtained at 50 °C, which decreased by 23.5% at 70 °C. Heating increased the molecular diffusion efficiency by reducing the system viscosity, but the hydrogen bond network of the DES component (octanoic acid-dodecanoic acid) might be dissociated due to thermal disturbance after more than 60 °C, resulting in the instability of the three-phase structure [40]. The abnormally high value of 0.0809 g at the 40 °C group suggests the possibility of uneven local heat distribution; however, the existing data still support 50 °C as the thermodynamically optimal condition.

When the top/bottom phase volume ratio was 1:1, the yield exceeded 0.0844 ± 0.0010 g (F = 35.18, *p* = 0.0003). The mass transfer at the phase interface was significantly regulated by the volume ratio: the 1:1 ratio could theoretically maximize the contact area and maintain a low interface energy barrier, while the volume expansion of the DES phase at >1.5:1 might cause eddy current disturbances and interfere with the directional migration of polysaccharides to the aqueous phase [41]. The sharp drop in yield (0.0801 g → 0.0706 g) at a volume ratio ≥1.5:1 confirmed the negative effect of fluid instability on mass transfer efficiency.

### 2.2. Optimization of TPP Extraction Process

#### 2.2.1. RSM Model Fitting

Based on the results of single-factor experiments, the effects of ammonium sulfate concentration, liquid–solid ratio, and top/bottom phase volume ratio on the yield of FCP were particularly significant. For these three key factors, the RSM was used to optimize the following parameters [42]: ammonium sulfate concentration (20, 25, 30 wt%), solid–liquid ratio (1:20, 1:25, 1:30 g/mL), and top/bottom phase volume ratio (0.5:1, 1:1, 1.5:1 *v*/*v*) were used as the response value. The experiment followed the principle of Box–Behnken three-factor and three-level design [43], and a total of 17 groups of experiments were carried out (the experimental scheme and results are shown in Table 1 and Table 2). Based on the experimental data, the quadratic polynomial regression model of FCP yield and the dependent variable was established as follows:

Y = 9.002 + 0.061A + 0.362B + 0.196C − 0.060AB + 0.002AC + 0.180BC + 0.742A^2^ − 1.590B^2^ − 0.752C^2^ (1)

Y was the yield of FCP (%); A, B, and C represented ammonium sulfate concentration (wt%), liquid–solid ratio (g/mL), and top/bottom phase volume ratio (*v*/*v*), respectively. Regression equation analysis of variance (ANOVA) results were shown in Table 2:

In terms of model significance, the regression equation was extremely significant (*p* < 0.0001), while the lack of fit term was not significant (*p* = 0.1119 > 0.05), indicating that the model could effectively predict the optimal conditions [44]. In terms of goodness of fit, the coefficient of determination R^2^ = 0.9902, the adjusted coefficient of determination R^2^_Adj_ = 0.9777, and the predicted R^2^ = 0.8800 confirmed that the model fit well and the prediction reliability was high. For the significance of each factor, the extremely significant factors include B^2^ (*p* < 0.0001), A^2^ (*p* < 0.0001), C^2^ (*p* < 0.0001), B (*p* = 0.0004), and AB (*p* = 0.0106). The significant factor was C (*p* = 0.0106). The non-significant factors included A (*p* = 0.3167), BC (*p* = 0.4795), and AC (*p* = 0.9760). According to the contribution of the F value, the order of the three factors on the yield of FCP was B^2^ > A^2^ > C^2^ > B > AB > C (Note: A, BC, and AC had no significant effect).

The model contained several key features. First, the quadratic term dominated: B^2^ (F = 412.10), A^2^ (F = 89.83), and C^2^ (F = 92.27) had extremely significant negative effects (coefficients were all negative), confirming that the three had an open downward parabolic effect on the yield (there was a maximum point). The strong negative effect of B^2^ (−1.590) indicated that when the volume ratio exceeded 1:1, the intensification of Marangoni convection led to a sharp drop in yield (such as the yield of 7.58% when Run 2: B = 1.5) [45]. In terms of interaction effects, AB had a significant negative interaction (−0.180, *p* = 0.0106): high volume ratio (B > 1) and high ammonium sulfate concentration (A > 25 %) synergistically destroyed the phase equilibrium (such as Run 10: B = 1.5/A = 30% yield 6.88%). There was no significant interaction between BC and AC (*p* > 0.05), which could be ignored. The contribution of the linear term was mainly due to the positive effect of B (0.362, *p* = 0.0004), and the volume ratio increased the yield in the range of 0.5:1–1:1 (9.14% at Run 1:B = 1). The model revealed the non-linear mechanism that was not captured by the single-factor experiment through significant quadratic terms (A^2^, B^2^, C^2^) and interaction terms (AB). It was predicted that the optimal conditions were a liquid–solid ratio of 1:24.2 g/mL, a volume ratio of 1:1.05 *v*/*v*, and an ammonium sulfate concentration of 25.8% (predicted yield of 9.27%), which provided a quantitative basis for the three-phase separation process.

#### 2.2.2. Interactive Effects of Factors

**Figure 2:**
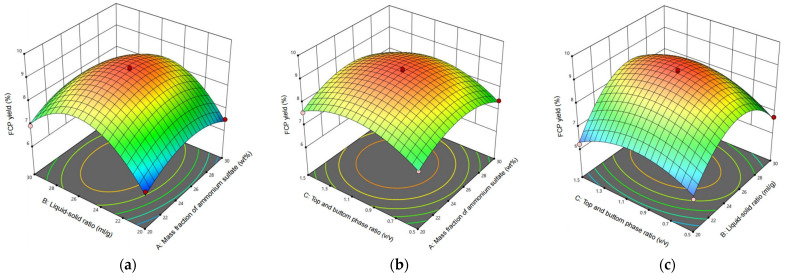
Three-dimensional response surface (**a**–**c**) showed the effect of interactions among three factors on the FCP yield. Part A is the ammonium sulfate concentration (wt%), part B is the liquid–solid ratio (*v*/*v*), and part C is the top and bottom phase volume ratio, respectively.

The complex interaction between multiple factors had a significant effect on the yield of FCP. The three-dimensional response surface composed of ammonium sulfate concentration (A), liquid–solid ratio (B), and top/bottom phase volume ratio (C) showed strong non-linear steep characteristics (Figure 2), which clearly showed that the interaction between the three factors played a decisive role in the extraction results [46]. It was worth noting that the interaction between the ammonium sulfate concentration (A) and the liquid–solid ratio (B) (AB) had a significant effect on the FCP yield (*p* = 0.0106), and its positive interaction effect (coefficient + 0.180) indicated that the combination of high volume ratio and low salt concentration could synergistically improve the mass transfer efficiency [47]. However, when ammonium sulfate concentration exceeded 25%, the increase in volume ratio would cause interfacial turbulence and lead to a sharp decrease of 15.2% in yield. The abrupt change region of the response surface diagram and the characteristics of the elliptical contour line were mutually confirmed with the variance analysis results, which together confirmed that the volume ratio (quadratic term coefficient −1.590) dominated the parabolic response by regulating the phase interface energy barrier. The model predicted that the optimal conditions were a liquid–solid ratio of 1:24.2 g/mL, a volume ratio of 1:1.05 *v*/*v*, and an ammonium sulfate concentration of 25.8%. At this time, the predicted FCP yield was 9.27%. The actual yield measured by the verification experiment was 9.22 ± 0.20%, and the deviation from the predicted value was only 0.53% (*t*-test *p* = 0.42). The high consistency between the measured value and the predicted value strongly proved the accuracy and reliability of the model [48].

### 2.3. Polysaccharide Yield and Purity Analysis

The yield, total sugar content, and protein content of FCP extracted under the optimal conditions are shown in Table 3. The yield of 9.22% conformed to the general characteristics of FCP yield and TPP technology; it was very close to the initial expected value (9.27%), and the difference was close to the experimental error [49], reflecting that the UA-DES-TPP system had good practical applicability. Compared with the traditional hot water extraction method in other studies (2.59 ± 0.01%), it still increased by 71.9%, confirming the destructive effect of DESs on the cell wall [50]. The main limiting factor lay in the fact that the intermediate phase formed by the salting out of ammonium sulfate might encapsulate partial polysaccharides [51]. The excellent purity with a total sugar content of 89.5% and a protein content of 1.8% mainly resulted from the directional separation effect at the DES/ammonium sulfate interface, where the formed protein precipitate layer physically blocked the sugar–protein co-extraction. Meanwhile, the 70 kDa dialysis membrane could effectively retain small-molecule impurities, and the high solubility of octanoic acid-dodecanoic acid (DES) for neutral polysaccharides and its low solubility for proteins also contributed to the purity to a certain extent.

### 2.4. Recycling and Reusing of DESs

**Figure 3:**
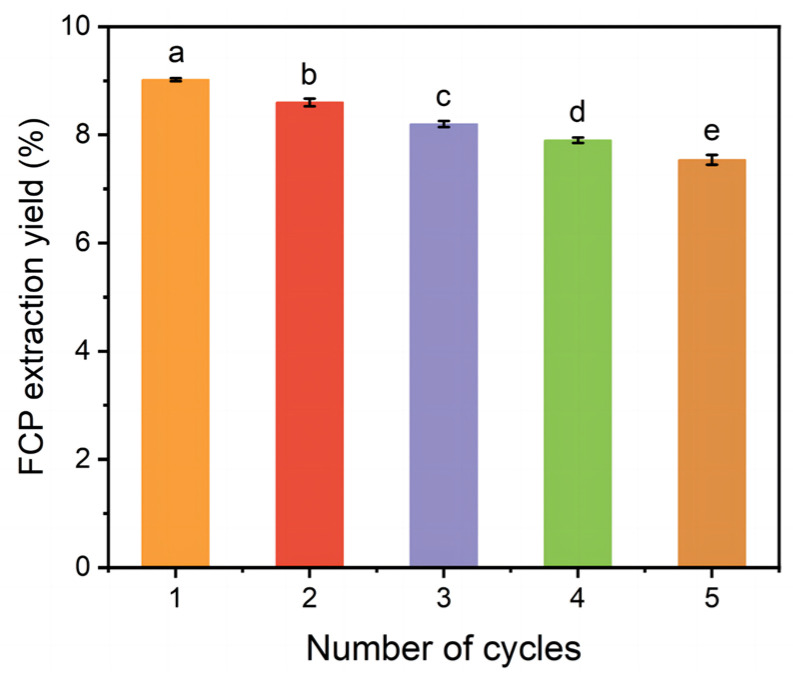
FCP extraction yield in recycling tests.

The cycle stability and recyclability of DESs were evaluated by the yield of FCP. The results were shown in Figure 3. After the first recovery, the yield of FCP was calculated to be 9.02 ± 0.02%, which was only 0.20% lower than that of the first extraction [52]. With the increase in the number of cycles, the yield of FCP gradually decreased. When the number of cycles reached five, the yield of FCP remained above 7%, indicating that the desorption system used in the TPP system had good cycle stability and recyclability [53].

### 2.5. Structural Characterization

#### 2.5.1. Scanning Electron Microscope Analysis

**Figure 4:**
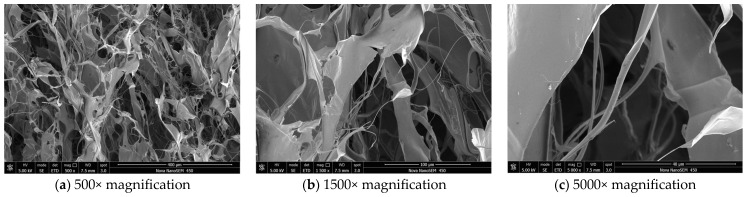
SEM micrographs of FCP at 500× (**a**), 1500× (**b**), and 5000× (**c**) magnifications.

The microstructure of FCP samples was visualized by scanning electron microscopy. The SEM images at 500×, 1500×, and 5000× magnifications are shown in Figure 4, respectively. At the lower 500× magnification, the overall microstructure of FCP could be clearly observed, showing relatively loose filamentous and reticulated structures, which indicated a disordered microstructure. At 1500× magnification, the morphology of the filamentous and reticular structures of FCP could be more clearly observed. At 5000× magnification, the polysaccharide microfibers in FCP showed a flat and straight morphology with a regular shape. This indicated that the TPP system did not destroy the polysaccharide network of FCP and enabled good preservation of the polysaccharide structure during the extraction process. Notably, no spherical or granular aggregates were observed at these magnifications, indicating the absence of proteins and the high purity of extracted FCP. These results demonstrated the effectiveness of TPP-DES extraction in effectively separating polysaccharides [54].

#### 2.5.2. Fourier Transform Infrared Spectrum Analysis

**Figure 5:**
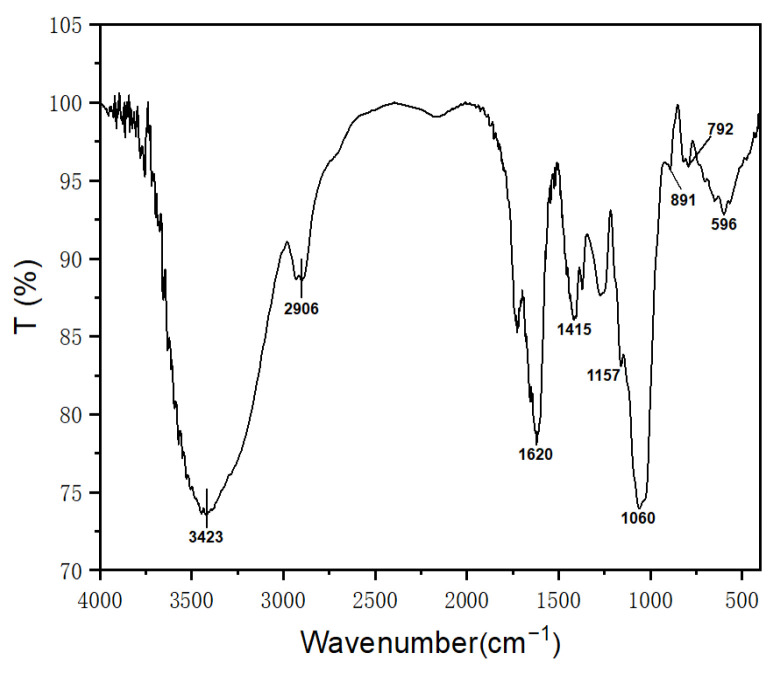
FT-IR spectrum of FCP.

FT-IR spectroscopy was used to investigate the molecular structure characteristics of FCP samples. The FT-IR spectra of FCP are shown in Figure 5. The prominent characteristic peak at 3423 cm^−1^ was caused by the O-H stretching vibration of hydroxyl groups, indicating the presence of hydrogen bonding within the polysaccharide. The weaker peak at 2906 cm^−1^ was attributed to the C-H stretching vibration. The band at 1620 cm^−1^ was caused by the C=O stretching vibration and hydrogen bonding coupled with COO, indicating the presence of bound water, while the peak at 1415 cm^−1^ indicated deformation vibration of the C-H bond. Two peaks in the region from 1000 to 1200 cm^−1^ indicated asymmetric stretching of C-O-C and C-O-H groups in the pyranose ring. Two characteristic peaks near 891 cm^−1^ and 792 cm^−1^ suggested the coexistence of α and β configurations of the pyranose, with the 891 cm^−1^ peak representing the β-configuration and the 792 cm^−1^ peak representing the α-configuration. Finally, the peak at 596 cm^−1^ showed the presence of pyran sugar rings.

#### 2.5.3. Monosaccharide Composition Analysis

**Figure 6:**
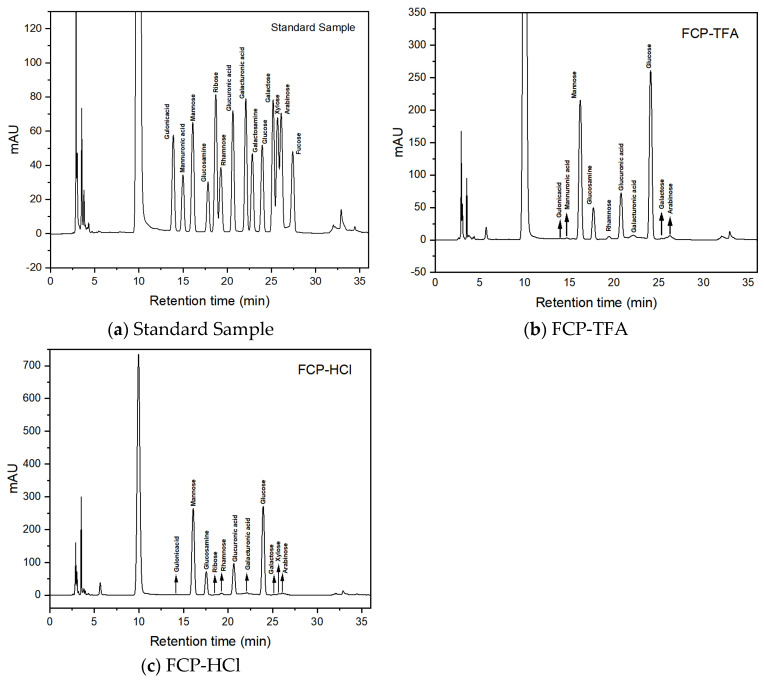
The monosaccharide composition of the standard sample (**a**), FCP-TFA (**b**), and FCP-HCl (**c**).

Previous studies had showed that bioactivities such as the antioxidant capacity of polysaccharides were influenced by monosaccharide composition. FCP trifluoroacetic acid hydrolyzed derivative (FCP-TFA), FCP hydrochloric acid hydrolyzed derivative (FCP-HCl), and monosaccharide standard were determined by the High Performance Liquid Chromatography (HPLC) technique. Figure 6 shows the chromatograms of standard, FCP-TFA, and FCP-HCl, respectively. The chromatogram of two derivatives showed that FCP was a typical heteropolysaccharide [55]. The chromatogram of FCP-TFA showed that the main components of FCP were glucose and mannose, as well as glucosamine, glucuronic acid, and trace amounts of arabinose, rhamnose, galacturonic acid, mannuronic acid, gluonic acid, and galactose. The contents of these monosaccharides were 277.779 mg/g, 181.292 mg/g, 84.689 mg/g, 55.163 mg/g, 6.879 mg/g, 5.411 mg/g, 3.830 mg/g, 2.953 mg/g, 0.948 mg/g, and 0.912 mg/g, respectively. From the peak areas, it could be seen that the monosaccharide molar ratio was 46.040:30.049:11.735:8.485:1.368:0.984:0.589:0.454:0.146:0.151 (See Table S1) [56]. The FCP-HCl chromatogram showed that the major components of FCP were glucose and mannose, along with glucosamine, glucuronic acid, and small amounts of rhamnose, arabinose, galacturonic acid, xylose, guluronic acid, galactose, and ribose. The content and molar ratio of these monosaccharides are shown in Table S2. The results suggested that the predominant constituents comprising the FCP sugar chain backbone were glucose and mannose.

### 2.6. Functional Properties

#### 2.6.1. Thermal Analysis

**Figure 7:**
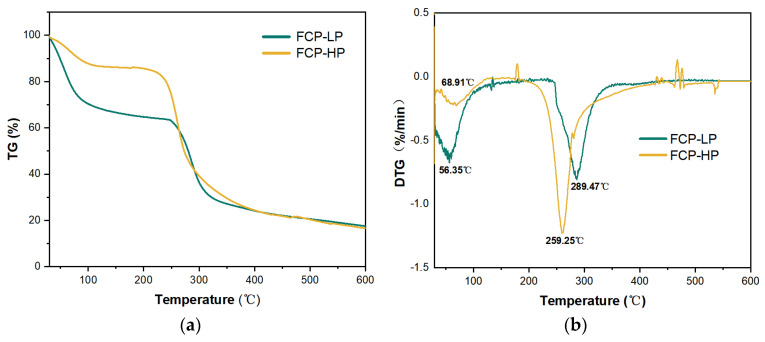
The TG (**a**) and DTG (**b**) curves of FCP.

The thermal properties of FCP were analyzed by thermogravimetric (TG) and derivative thermogravimetric (DTG) curves (Figure 7). The TG curves of low-purity FCP (FCP-LP) and high-purity FCP (FCP-HP) were similar in that there were three different stages of mass loss. The temperature intervals were in the range of 30–250 °C, 250–400 °C, and 400–600 °C, respectively [57]. They corresponded to the evaporation of free and bound water, thermal decomposition of polysaccharides, and combustion of carbon residues, respectively [58]. According to the DTG curves, FCP-HP was more susceptible to weight loss than FCP-LP at higher temperatures, which indicated that FCP-HP had superior thermal stability [59]. In addition, FCP-HP remained stable below 200 °C, indicating that it had good heat resistance in thermal processing. These results might be attributed to the ordered microstructure of high-purity FCP.

#### 2.6.2. Antioxidant Potentials

**Figure 8:**
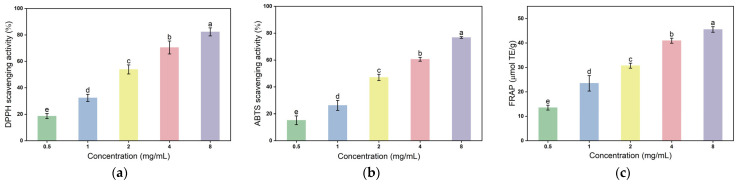
Variation in DPPH radical-scavenging percentage with varying concentrations for FCP (**a**), variation in ABTS radical-scavenging percentage with varying concentrations for FCP (**b**), and the antioxidant capacity of FRAP (**c**). The data were calculated from three parallel experiments.

The FCP showed significant concentration-dependent antioxidant activity in DPPH free radical scavenging, ABTS free radical scavenging, and FRAP reducing power tests (Figure 8). The comprehensive data of three in vitro experiments confirmed that it had excellent free radical neutralization ability. In the DPPH free radical scavenging experiment, the scavenging rate of FCP increased steadily from 18.6 ± 2.1% at 0.5 mg/mL to 82.3 ± 3.8% at 8 mg/mL, and the scavenging kinetics showed that the concentration ≥ 2 mg/mL had exceeded the 50% scavenging threshold (53.9 ± 4.2%), indicating that it had strong electron donating ability [60]. At the same time, the ABTS free radical scavenging test showed a similar trend: the scavenging rate increased significantly from 15.2 ± 3.8% (0.5 mg/mL) to 76.8 ± 0.8% (8 mg/mL), and the EC_50_ value was about 1.95 mg/mL, which further confirmed the efficient scavenging characteristics of FCP on various free radicals. It was worth noting that the ferric reducing antioxidant power (FRAP) test results showed that FCP exhibited a reducing power of 13.53 ± 0.85 μmol TE g^−1^ at a concentration of 0.5 mg/mL and increased linearly with the increase in concentration (R^2^ = 0.994). The reducing power reached 45.53 ± 1.07 μmol TE g^−1^ at the highest concentration of 8 mg/mL [61]. The three sets of parallel experimental data were highly consistent (standard deviation < 3.5%). This broad-spectrum antioxidant activity mainly stemmed from the continuous release of electrons by the abundant phenolic hydroxyl functional groups in FCP molecules as the concentration increases, as well as the unique polysaccharide chain conformation (such as α-glycosidic bonds) that enhanced the exposure of active sites [62]. This enabled a scavenging efficiency of over 20% and a reducing power of more than 23 μmol TE g^−1^ in the low concentration range (1–2 mg/mL). FCP synergistically exerts free radical scavenging and metal ion reduction functions through a concentration-dependent electron transfer mechanism. Its highly efficient and stable antioxidant properties provided a structural and functional basis for the development of natural antioxidants.

## 3. Materials and Methods

### 3.1. Materials and Reagents

Lyophilized *F. carica* was purchased from Weihai, Shandong. Petroleum ether and dodecanoic acid (hydrogen bond acceptor) were purchased from Xilong Science Co., Ltd. (Shantou, Guangdong, China). Octanoic acid (hydrogen bond donor) and ammonium sulfate (≥99%, used to construct a three-phase system) were provided by Tianjin Kemiou Chemical Reagent Co., Ltd. (Tianjin, China). Fourteen monosaccharide standards for HPLC analysis (gulonic acid, mannuronic acid, mannose, glucosamine, ribose, rhamnose, glucuronic acid, galacturonic acid, galactosamine, glucose, galactose, xylose, arabinose, and fucose), acetonitrile (HPLC mobile phase), trifluoroacetic acid (TFA, ≥99%, polysaccharide hydrolysis), D-glucose, phenol (determination of polysaccharide content by phenol-sulfuric acid method), and bovine serum albumin (BSA, determination of protein by Coomassie brilliant blue method) were provided by Shanghai McLean Biochemical Technology Co., Ltd. (Shanghai, China). DPPH (2,2-diphenyl-1-picrylhydrazyl radical), ABTS (2,2-azinobis-3-ethylbenzothiazoline-6-sulfonic acid), FRAP working solution (containing TPTZ, Fe^3+^, and acetic acid buffer), and Trolox (water-soluble vitamin E analogs, standard curve establishment) were purchased from Fuzhou Feijing Biotechnology Co., Ltd. (Fuzhou, Fujian, China). Sodium phosphate buffer (HPLC mobile phase) provided by Hangzhou Xinran Biotechnology Co., Ltd. (Hangzhou, Zhejiang, China).

### 3.2. Ultrasound-Assisted Deep Eutectic Solvents-Based Three-Phase Partitioning (UA-DES-TPP) for FCP Extraction

#### 3.2.1. Pretreatment of Freeze-Dried *F. carica*

Lyophilized *F. carica* was ground to a fine powder by a grinder (BAIJIE-800A, Baijie, Hangzhou, China) and passed through a 60-mesh sieve. The powder and petroleum ether were loaded into a Soxhlet extractor at a liquid–solid ratio of 10:1 and heated and refluxed in a 40 °C thermostatic water bath for 2 h to fully degrease. The degreased powder was placed in a constant temperature drying oven (DZF-6020MBE, Boxun, Shanghai, China); the temperature was set at 35 °C, the ventilation mode was turned on, and the surface petroleum ether was removed after drying for 18 h. The collected powder samples were dried at a constant temperature and stored for later use.

#### 3.2.2. Preparation of DESs

The preparation method of DESs for polysaccharide extraction was as follows: HBA and HBD were mixed in a specific molar ratio, and the mixture was continuously magnetically stirred in a constant temperature water bath at 80 °C to form a uniform transparent liquid without visible particles or stratification. The DES, composed of dodecanoic acid and octanoic acid (1:1 molar ratio), was selected based on its favorable physicochemical properties, including low viscosity and suitable hydrophobicity, which facilitate efficient phase separation and polysaccharide extraction. This combination has been reported to achieve high extraction yields in similar systems [63,64]. The HBA was dodecanoic acid, and the HBD was octanoic acid.

#### 3.2.3. FCP Extraction

The lyophilized *F. carica* powder (1.0 g) was accurately weighed and mixed with a determined DES (dodecanoic acid and octanoic acid with a molar ratio of 1:1). Ammonium sulfate solution was added to construct a TPP system. The specific ammonium sulfate solution preparation parameters are shown in Table 4. The extraction parameters were set as follows: temperature, ultrasonic power, and time. After extraction, the mixture was centrifuged (5000 rpm × 15 min) to form three layers: the upper DES phase, the middle protein precipitation, and the lower water phase composed of ammonium sulfate solution and polysaccharide. After collecting the lower components, the dialysate was dialyzed for 48 h by a 70 kDa retention dialysis bag in flowing deionized water, and the dialysate was freeze-dried to obtain a polysaccharide sample. The yield of polysaccharides extracted from each DES system was determined by the phenol–sulfuric acid method combined with a glucose standard curve.

### 3.3. Single-Factor Experiments of FCP Extraction

Under the premise of selected DES and fixed ultrasonic settings, the variables affecting the extraction of UA-DES-TPP include extraction time, extraction temperature, ammonium sulfate content, liquid–solid ratio, and top/bottom phase volume ratio. In order to determine the optimal conditions for ultrasonic-assisted DES extraction of FCP. The effects of extraction time (10 min, 20 min, 30 min, 40 min, 50 min), extraction temperature (30 °C, 40 °C, 50 °C, 60 °C, 70 °C), ammonium sulfate mass fraction (15%, 20%, 25%, 30%, 35%), liquid–solid ratio (1:10, 1:15, 1:20, 1:25, 1:30), and top/bottom phase volume ratio (0.5:1, 1:1, 1:1, 1.5:1, 2:1, 2:1, 2:1, 2.5:1) on the yield of FCP were studied by single-factor experiment [65].

### 3.4. Response Surface Optimization of FCP Extraction

RSM and a second-order polynomial were used to further optimize the extraction conditions. Based on the results of single-factor experiments, three key influencing factors of ammonium sulfate mass fraction (A), liquid–solid ratio (B) and top/bottom phase volume ratio (C) were selected as independent variables in Box–Behnken design (BBD), and three levels (1, 0, −1) were set for each factor. A total of 17 groups of experiments were carried out, and the FCP yield was used as the response value. The experimental design of three factors and three levels is shown in Table S3. Then, the optimal extraction process was determined by regression analysis, and the actual experiment was carried out under the determined conditions to verify the effectiveness of the established model [66].

### 3.5. Yield and Purity

#### 3.5.1. Standard Curve Establishment

The glucose standard curve by phenol–sulphuric acid assay was measured using glucose standard (0–100 μg/mL) at 490 nm (Figure S1), and the linear regression equation obtained was as follows:

y = 0.0058x + 0.0231 (R^2^ = 0.9984) (2)

where y represented the absorbance and x represented the glucose concentration (μg/mL).

The protein standard curve by Bradford assay was measured using BSA standards (0–100 μg/mL) at 595 nm (Figure S1), and the linear regression equation obtained was as follows:

y = 0.0062x + 0.0177 (R^2^ = 0.9992) (3)

where y represented the absorbance and x represented the protein concentration (μg/mL).

#### 3.5.2. Polysaccharide Yield and Purity

The total sugar content was determined by the phenol–sulfuric acid method with D-glucose as standard. According to the standard curve of bovine serum albumin, the protein content was determined by the Coomassie brilliant blue method, and the purity of the polysaccharide was evaluated [67]. The yield of FCP was calculated according to the following formula:

(4) $$FCP\;yield\;(\%)=\frac{FCP\;mass\;(g)}{dried\;fig\;powder\;mass\;(g)}\times 100\%$$

### 3.6. Recycling and Reusing of DESs

The recovery and reuse experiments were designed to evaluate the reusability of the DES solvent after FCP extraction. After each FCP extraction, the upper DES phase was separated, collected, and used for the next TPP extraction without further purification. Repeat tests were performed for five cycles. If the recovered DES could still extract a certain amount of polysaccharide, then it was deemed recoverable, indicating that its performance was not significantly impaired [68].

### 3.7. Structural Characterization

#### 3.7.1. Scanning Electron Microscope (SEM)

The lyophilized FCP sample was immobilized on a sample stage for SEM. Subsequently, the samples were coated with a thin layer of gold to improve the conductivity for easy observation, and the surface microstructures of the FCP samples were observed using a field emission-scanning electron microscope (Nano SEM 450, FEI Ltd., Hillsboro, OR, USA) at a 20 kV accelerating voltage [69].

#### 3.7.2. Fourier Transform Infrared Spectrum (FT-IR)

The FCP samples were thoroughly mixed with dry potassium bromide powder at a ratio of 1:100 and uniformly ground before being pressed into thin films. Subsequently, a full-range scan was conducted using an FT-IR spectrophotometer (TENSOR27, Bruker, Bremen, Germany) with a resolution of 4 cm^−1^ and a range of 4000–400 cm^−1^ [70].

#### 3.7.3. Monosaccharide Composition

Monosaccharide content was measured by high-performance liquid chromatography (HPLC) (Agilent 1100, Thermo Fisher, Waltham, MA, USA). The 14 monosaccharide standards—gulonic acid, mannuronic acid, mannose, glucosamine, ribose, rhamnose, glucuronic acid, galacturonic acid, galactosamine, glucose, galactose, xylose, arabinose, and fucose—were prepared into a monosaccharide mixed standard solution, and the mass concentration of each monosaccharide was 0.4 mg/mL. A total of 15 mg of FCP samples was hydrolyzed in a 10 mL hydrolysis tube with 5 mL 2 mol/L trifluoroacetic acid (TFA) at 110 °C for 2 h. Then 1 mL of the solution was mixed with methanol at a ratio of 1:1 and dried with N_2_ in a 70 °C water bath, repeated twice to remove TFA. Subsequently, 1 mL of 0.3 mol/L NaOH solution was added to fully dissolve the residue, which was the polysaccharide hydrolysate. A total of 400 μL of monosaccharide mixed standard solution or polysaccharide hydrolysate was taken in a 5 mL plug test tube. A total of 400 μL of PMP methanol solution was added and vortexed, and the reaction was carried out in a water bath at 70 °C for 2 h. Take out and cool to room temperature, add 400 μL 0.3 mol/L HCl to neutralize the pH to about 7. Add 1200 μL of water and an equal volume of chloroform, vortex mixing, shake after standing, and remove the chloroform phase, then extract twice. The aqueous phase was filtered with a 0.45 μm microporous membrane for HPLC analysis. The parameters of HPLC analysis were as follows: chromatographic column, Agilent C18 (4.6 × 250 nm, particle size of 5 μm); detector, DAD detector; mobile phase A, 100 mM sodium phosphate buffer (pH = 6.7); mobile phase B, acetonitrile; detection wavelength, 250 nm; flow rate, 1 mL/min; injection volume, 5 μL; column temperature, 30 °C. The mobile phase gradient system conditions were: 0–9 min, A:B was 86:14; 9.1–28 min, A:B was 83:17; 28.1–29 min, A:B was 78:22; 29.1–32 min, A:B = 50:50; 32.1–36 min, A:B was 86:14 [71].

### 3.8. Functional Properties

#### 3.8.1. Thermal Stability

The thermodynamic properties of FCP samples were characterized using a thermogravimetric analyzer (1600LF, METTLER, Greifensee, Switzerland). The measurements were carried out in a nitrogen atmosphere (flow rate of 50 mL/min) over a temperature range of 30–600 °C with a heating rate of 10 °C/min. The percentage weight loss of FCP was determined from the TG curve, and the maximum decomposition temperature was determined from the DTG curve [72].

#### 3.8.2. Evaluation of Antioxidant Activity

The antioxidant properties were evaluated using the DPPH and ABTS free radical scavenging activity methods and the Ferric Reducing Antioxidant Power (FRAP). FCP samples (100 mg) were dissolved in 10 mL of 95% ethanol. The mixture was stirred under light-protected conditions for 24 h, resulting in the solution being the mother solution. Then, the mother solution was gradually diluted to obtain sample solutions of 0.5, 1, 2, 4, and 8 mg/mL [73]. Subsequently, 100 µL of DPPH ethanol solution (0.2 mmol/L) and 100 µL of the sample solution were added to a 96-well plate and allowed to react in the dark for 30 min. The absorbance was then measured at 517 nm (A_g_). Then, 100 µL of the sample solution was mixed with 100 µL of 95% ethanol solution, yielding A_C_. Additionally, 100 µL of DPPH ethanol solution was mixed with 100 µL of 95% ethanol solution, and the absorbance was measured as A_O_. The DPPH radical scavenging activity was calculated as follows:

(5) $$DPPH\;scavenging\;rate\;(\%)=(1-\frac{A_{g}-A_{C}}{A_{O}})\times 100\%$$

A total of 180 µL of the ABTS solution was mixed with 20 µL of the sample solution and allowed to react at room temperature in the dark for 6 min. Subsequently, the absorbance was measured at 734 nm, denoted as A_1_. A total of 20 µL of PBS solution was used in place of the sample solution to obtain the absorbance as the control, referred to as A_2_. Additionally, 180 µL of PBS solution was substituted for the ABTS solution to measure the absorbance, designated as A_0_. The ABTS radical activity is calculated as follows:

(6) $$ABTS\;scavenging\;rate\;(\%)=(1-\frac{A_{1}-A_{0}}{A_{2}})\times 100\%$$

The acetic acid buffer (pH 3.6), 10 mM 2,4,6-tripyridyl-3-azide (TPTZ), and 20 mM Fe^3+^ solution were mixed at a ratio of 10:1:1 and diluted 50 times with ethanol to prepare the FRAP working solution. The standard curve was constructed using Trolox. The FCP sample solution was mixed with the FRAP working solution and incubated at 37 °C in the dark. The absorbance was measured at 593 nm and recorded as A_a_. The absorbance obtained by replacing the sample solution with ethanol was recorded as A_b_. By referring to the standard curve, the antioxidant capacity was expressed by Trolox equivalent antioxidant concentration (TEAC). The calculation is as follows:

(7) $$FRAP\;capability\;(\mu mol\;TE/g)=(\frac{\left(A_{a}-A_{b}\right)}{Slope\;of\;the\;standard\;curve})\times 100\%$$

### 3.9. Statistical Analysis

All experiments were conducted in triplicate in this work, and the data were presented as mean ± standard deviation (mean ± SD). Statistical analysis was carried out using Origin 2022. Statistical differences were determined by one-way ANOVA, with statistical significance denoted by *p* < 0.05.

## 4. Conclusions

This study developed an efficient and green technology of UA-TPP-DES for the extraction and purification of FCP. The DES, composed of dodecanoic acid and octanoic acid (1:1 molar ratio), successfully addresses the environmental issues related to the traditional solvent tert-butanol. The optimal extraction process was determined through single-factor experiments and response surface methodology: liquid-to-solid ratio 1:24.2 g/mL, top phase/bottom phase volume ratio 1:1.05 *v*/*v*, ammonium sulfate concentration 25.8%, ultrasonic time 50 min, and 50 °C. Under these conditions, the polysaccharide yield reached 9.22 ± 0.20%. Moreover, DESs demonstrated excellent recyclability, with only a 1.48 ± 0.10% reduction in extraction rate after five cycles. The main monosaccharide composition of the obtained FCP was glucose and mannose. Functional property tests showed that FCP had excellent thermal stability and antioxidant activity. At a concentration of 8 mg/mL, the DPPH and ABTS radical scavenging rates reached 82.3 ± 3.8% and 76.8 ± 0.8%, respectively, and the FRAP antioxidant capacity was 45.53 ± 1.07 µmol TE g^−1^. This technology not only significantly improves the extraction efficiency and product quality but also provides a sustainable solution for the large-scale green production of *F. carica* polysaccharides and the development of functional foods.

## Figures and Tables

**Table 1:** Box–Behnken response surface design and corresponding response values.

Run	Liquid–Solid Ratio (g/mL)	Top Phase to the Bottom Phase Ratio (*v*/*v*)	Mass Fraction of (NH_4_)_2_SO_4_ (wt%)	Yield (%)
1	25	1	25	9.14
2	25	1.5	20	7.58
3	20	1	20	6.34
4	25	1	25	8.89
5	30	1.5	30	7.77
6	25	1.5	25	6.22
7	30	0.5	30	7.43
8	25	1	25	8.96
9	25	1	25	8.93
10	25	1	25	9.09
11	20	1	20	6.94
12	30	1	30	6.52
13	25	0.5	25	6.13
14	20	0.5	20	7.25
15	30	1	30	6.88
16	25	1.5	25	7.55
17	25	0.5	25	6.74

**Table 2:** ANOVA analysis of the quadratic model.

Source	Sum of Squares	df	Mean Square	F Value	*p*-Value	Significance
Model	18.3500	9	2.0400	78.9500	< 0.0001	**
A	0.0300	1	0.0300	1.1600	0.3167	ns
B	1.0500	1	1.0500	40.7100	0.0004	**
C	0.3081	1	0.3081	11.9300	0.0106	*
AB	0.0144	1	0.0144	0.5577	0.4795	ns
AC	0.0000	1	0.0000	0.0010	0.9760	ns
BC	0.1296	1	0.1296	5.0200	0.0601	ns
A^2^	2.3200	1	2.3200	89.8300	< 0.0001	**
B^2^	10.6400	1	10.6400	412.1000	< 0.0001	**
C^2^	2.3800	1	2.3800	92.2700	< 0.0001	**
Residual	0.1808	7	0.0258			
Lack of	0.1345	3	0.0448	3.8700	0.1119	ns
fit						
Pure	0.0463	4	0.0116			
error						
Cor. total	18.5300	16				
R^2^	0.9902					
R^2^_Adj_	0.9777					
Pred. R^2^	0.8800					

The symbols * and ** indicate significant differences at *p* < 0.05 and *p* < 0.01, respectively. The ns indicates no significance.

**Table 3:** The yield, sugar content, and protein content of FCP prepared through UA-DES-TPP extraction.

	Testing Value (%)	RSD (n = 3)
FCP extraction yield	9.22 ± 0.20	2.27%
Sugar content	89.50 ± 0.50	0.56%
Protein content	1.80 ± 0.10	5.56%

**Table 4:** Preparation parameters of 50 mL ammonium sulfate solution with five concentrations (base temperature 25 °C).

Parameter	15%	20%	25%	30%	35%
Density (g/cm^3^)	1.103	1.124	1.148	1.168	1.193
Theoretical total mass (g)	55.150	56.200	57.400	58.400	59.650
Mass of ammonium sulfate (g)	8.273	11.240	14.350	17.520	20.878
Ultra-pure water quality (g)	46.877	44.960	43.050	40.880	38.772

## Data Availability

The data presented in this study are available from the corresponding author upon request.

## Supplementary Materials

The following supporting information can be downloaded at https://www.mdpi.com/article/10.3390/molecules30173469/s1: Figure S1: The standard curve of glucose and protein; Table S1: The monosaccharide composition of FCP-TFA; Table S2: The monosaccharide composition of FCP-HCl; Table S3: Factors and levels for Box–Behnken center combination experimental design.
