# Supplementary material for: Extraction of Ficus carica Polysaccharide by Ultrasound-Assisted Deep Eutectic Solvent-Based Three-Phase Partitioning System: Process Optimization, Partial Structure Characterization, and Antioxidant Properties
Source: Molecules. 2025 Aug 23;30(17):3469. doi: 10.3390/molecules30173469 (PMC12430428; doi:10.3390/molecules30173469)
Supplement: Supplementary file 1 [file molecules-30-03469-s001.zip › molecules-3803647-supplementary.pdf]

Supporting Information for

"Extraction of ficus carica polysaccharide by ultrasound-assisted deepeutectic solvents-based three-phase partitioning system: process optimization, partial structure characterization and antioxidant properties"

Qisen Sun [1], Zhubin Song [1], Fanghao Li [1], Xinyu Zhu [1], Xinyu Zhang [2] and Hao Chen [2,*]

Contents:

1. Figure S1. The standard curve of glucose and protein.

2. Table S1. The monosaccharide composition of FCP-TFA.

3. Table S2. The monosaccharide composition of FCP-HCl.

4. Table S3. Factors and levels for Box Behnken center combination experimental design.

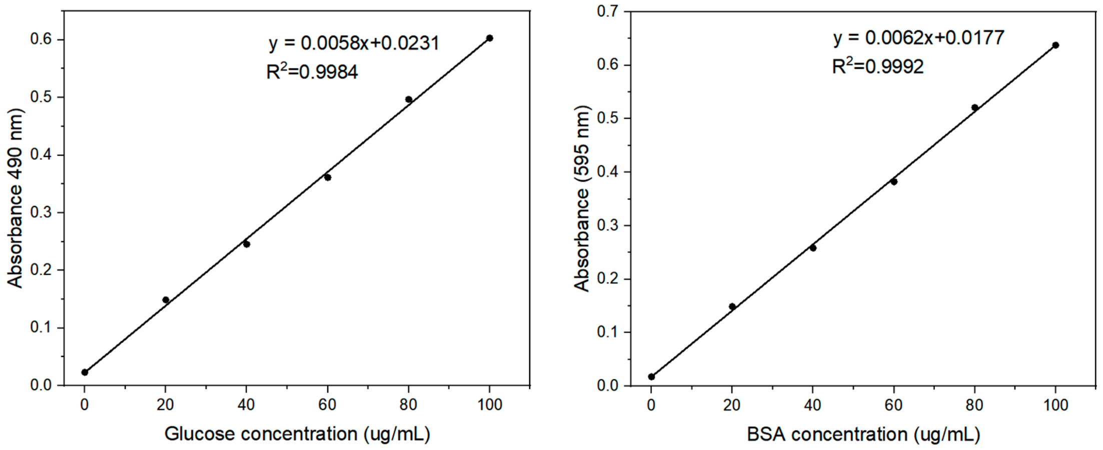

(**a**). The glucose standard curve (**b**). The protein standard curve

1. Figure S1. The standard curve of glucose and protein.

2. Table S1. The monosaccharide composition of FCP-TFA.

Monosaccharide composition	Retention time(min)	Peak area(pA s)	Content (mg/g)	Molar ratio%
Gulonicacid	13.949	19.294	0.948	0.146
Mannuronic acid	14.704	36.313	2.953	0.454
Mannose	16.208	4438.300	181.292	30.049
Glucosamine	17.677	900.614	84.689	11.735
Ribose	0.000	0.000	0.000	0.000
Rhamnose	19.437	82.878	5.411	0.984
Glucuronic acid	20.766	1392.410	55.163	8.485
Galacturonic acid	22.148	104.915	3.830	0.589
Galactosamine	0.000	0.000	0.000	0.000
Glucose	24.0698	5391.09	277.779	46.040
Galactose	25.281	25.311	0.912	0.151
Xylose	0.000	0.000	0.000	0.000
Arabinose	26.209	178.556	6.879	1.368
Fucose	0.000	0.000	0.000	0.000

3. Table S2. The monosaccharide composition of FCP-HCl.

Monosaccharide composition	Retention time(min)	Peak area(pA s)	Content (mg/g)	Molar ratio%
Gulonicacid	14.156	29.238	1.422	0.204
Mannuronic acid	0.000	0.000	0.000	0.000
Mannose	16.083	5030.150	203.392	31.489
Glucosamine	17.552	1199.460	111.652	14.451
Ribose	18.712	6.852	0.216	0.040
Rhamnose	19.262	96.540	6.239	1.060
Glucuronic acid	20.640	1692.980	66.394	9.539
Galacturonic acid	22.107	139.526	5.043	0.724
Galactosamine	0.000	0.000	0.000	0.000
Glucose	23.932	5171.300	263.763	40.835
Galactose	25.159	26.619	0.949	0.147
Xylose	25.696	53.639	2.150	0.399
Arabinose	26.085	156.917	5.984	1.112
Fucose	0.000	0.000	0.000	0.000

4. Table S3. Factors and levels for Box Behnken center combination experimental design.

Levels	Factors		
	A	B	C
	Liquid-solid ratio	Volume ratio of the top phase to the bottom phase	Mass fraction of $(NH_4)_2SO_4$
-1	1:20	0.5:1	20%
0	1:25	1:1	25%
1	1:30	1.5:1	30%
